# In Vitro Release Study of the Polymeric Drug Nanoparticles: Development and Validation of a Novel Method

**DOI:** 10.3390/pharmaceutics12080732

**Published:** 2020-08-04

**Authors:** Jingwen Weng, Henry H. Y. Tong, Shing Fung Chow

**Affiliations:** 1 Department of Pharmacology and Pharmacy, Li Ka Shing Faculty of Medicine, The University of Hong Kong, Pokfulam, Hong Kong SAR, China; irenewjw@connect.hku.hk; 2 School of Health Sciences and Sports, Macao Polytechnic Institute, Macao SAR, China; henrytong@ipm.edu.mo

**Keywords:** polymeric nanoparticle, in vitro release kinetics, centrifugal ultrafiltration, dialysis, filtration

## Abstract

The in vitro release study is a critical test to assess the safety, efficacy, and quality of nanoparticle-based drug delivery systems, but there is no compendial or regulatory standard. The variety of testing methods makes direct comparison among different systems difficult. We herein proposed a novel sample and separate (SS) method by combining the United States Pharmacopeia (USP) apparatus II (paddle) with well-validated centrifugal ultrafiltration (CU) technique that efficiently separated the free drug from nanoparticles. Polymeric drug nanoparticles were prepared by using a four-stream multi-inlet vortex mixer with d-α-tocopheryl polyethylene glycol 1000 succinate as a stabilizer. Itraconazole, cholecalciferol, and flurbiprofen were selected to produce three different nanoparticles with particle size <100 nm. By comparing with the dialysis membrane (DM) method and the SS methods using syringe filters, this novel SS + CU technique was considered the most appropriate in terms of the accuracy and repeatability to provide the in vitro release kinetics of nanoparticles. Interestingly, the DM method appeared to misestimate the release kinetics of nanoparticles through separate mechanisms. This work offers a superior analytical technique for studying in vitro drug release from polymeric nanoparticles, which could benefit the future development of in vitro-in vivo correlation of polymeric nanoparticles.

## 1. Introduction

Recent advances in nanotechnology have revolutionized the administration of drugs to different organs for a wide array of diseases. Compared to conventional dosage forms, drug-loaded nanoparticles possess multiple pharmaceutical merits, including improved solubility, protection of cargo, sustained drug release, and targeted delivery that can increase the therapeutic index of medicines [1,2]. Despite these promising potentials, only 51 nanomedicines have been approved by the United States Food and Drug Administration (US FDA) till 2018 [3] One of the major hurdles for getting the approval is the lack of specific regulatory guidelines. According to the FDA guidelines, most of the nanoparticle-based pharmaceutical products still follow the typical drug development process, which has been highly criticized [4,5]. The insufficiency of standardized testing protocols to evaluate the in vitro and in vivo properties of nanomedicines at the early stage may cause unpredictable therapeutic outcome and thus a high risk of failure in clinical trials, impeding the clinical translation of nanoparticle-based drug delivery systems.

Among all the in vitro tests for nanoparticles, the release study is one of the controversial experiments. The in vitro release kinetics of nanoparticles provides critical information regarding their ability to modify the drug release, thus is an important parameter to be considered for the assessment of the safety, efficacy, and quality of the products. When performed properly, they can be correlated to in vivo behaviors through predictive mathematical models [6,7], leading to an acceleration of regulatory approval. In the past decade, workshops held by the American Association of Pharmaceutical Scientists (AAPS) and the International Pharmaceutical Federation (FIP) have reached a consensus that appropriate considerations should be taken before a release method can be recommended to a novel dosage form like nanoparticles [8,9].

There are no compendial or regulatory standards thus far. A review on literature indicates that the drug release profiles of nanoparticle-based formulations can be obtained by three methods, namely continuous flow (CF), dialysis membrane (DM), and sample and separate (SS) methods [10]. In the CF method, USP apparatus IV is employed and the literature on its application to the in vitro release study of nanoparticles is sparse. It is costly and also difficult to set up and maintain a constant flow rate [11]. The DM method is the most popular method to test the in vitro release kinetics of nanoparticles. Irrespective of the different set-ups, they all rely on a semi-permeable membrane to achieve physical separation of the nanoparticle and the free drug. However, the drug concentration in the receiver compartment may not timely reflect the actual concentration of the free drug release from the nanoparticle [12,13,14]. The SS method is another commonly used approach to assess the release kinetics. Most of the researches employed unstandardized equipment as the vessel, such as the polypropylene tube and the glass beaker, which made the testing conditions difficult to be kept consistent [15,16]. Also, a complete physical separation of nanoparticles and free drug is challenging because of the tiny size of nanoparticles. The separation techniques vary from the traditional syringe filters [17] to newly developed techniques like ultracentrifugation [18], pressure ultrafiltration [16], and centrifugal ultrafiltration [19]. Nevertheless, these recently developed techniques were rarely applied to the release study, but employed to determine the drug loading and encapsulation efficiency.

**Figure 1:**
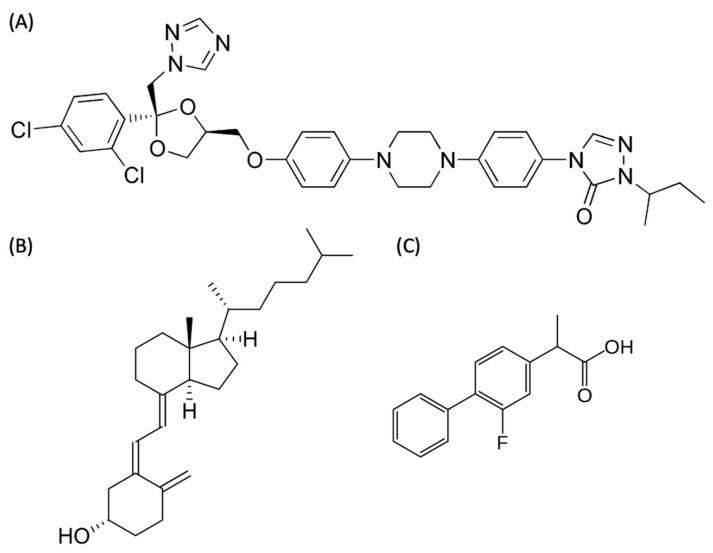
Chemical structures of (**A**) itraconazole (ITZ), (**B**) cholecalciferol (VitD3), and (**C**) flurbiprofen (FLU).

There is an imperative need for the development of an appropriate method studying the in vitro release kinetics of nanoparticles. As the polymeric nanoparticle is the mainstay of the nanoparticle type because of its extensive medical applications, high biocompatibility, and relatively stable drug release, we herein utilized a four-stream multi-inlet vortex mixer (MIVM) to prepare different polymeric drug nanoparticles based on the principle of flash nanoprecipitation [20]. D-α-tocopheryl polyethylene glycol 1000 succinate (TPGS) was selected as the stabilizer because it is a biodegradable PEGylated polymer approved by the FDA as a safe excipient. Three model drug compounds with poor aqueous solubilities, namely itraconazole (ITZ), cholecalciferol (VitD3), and flurbiprofen (FLU), were formulated as nanoparticles for method comparison (Figure 1 and Table 1). ITZ is a weak base (pK_a_ = 3.7) [21] with high lipophilicity (log *P* = 5.7) [22] and is not soluble in water (<0.09 μg/mL). VitD3 is non-ionic and practically water-insoluble (<0.05 μg/mL) which possesses the highest lipophilicity among the three model drugs (log *P* = 7.5) [23]. FLU has the highest aqueous solubility (22.0 μg/mL) among the three compounds but is still considered as a weak acid (pK_a_ = 4.5) [24] and lipophilic (log *P* = 4.2) [25].

In this study, we developed and validated a novel SS method by combining USP apparatus II (paddle) and centrifugal ultrafiltration, comparing with the DM method and the traditional SS methods using two different sized syringe filters (0.45 and 0.2 µm). By using the USP apparatus II (paddle), the control of the testing conditions such as temperature, mode of agitation, and sampling technique becomes straightforward. Also, the centrifugal ultrafiltration technique is capable of separating the free drug from the nanoparticles efficiently. Regardless of the test methods, a sink condition was used, under which the volume of the release medium should be three times larger than that required to obtain a saturated drug solution [26]. Hence, the solubilities of model drugs in their FDA recommended release medium were determined prior to the release study. In addition, the drug loss during separation techniques was tested and the centrifugal ultrafiltration technique was validated. In order to interpret the release kinetics obtained, mathematical modeling was further applied for comparative analysis. In overall, the goals of this study were to (1) validate the centrifugal ultrafiltration technique, (2) study and compare the four different analytical methods for the in vitro release study, (3) identify and compare the release mechanisms by fitting the data into the two well-established mathematical models.

## 2. Material and Methods

### 2.1. Materials

Itraconazole (ITZ) and flurbiprofen (FLU) were supplied from Yick Vic Chemicals & Pharmaceuticals Ltd (Hong Kong SAR, China). Cholecalciferol (VitD3), cholesterol (CLT), d-α-Tocopherol polyethylene glycol 1000 succinate (TPGS), poly(vinyl alcohol) (PVA, average MW 13,000–23,000), trifluoroacetic acid, ammonium acetate, and sodium dodecyl sulfate (SDS) were purchased from Sigma Aldrich (St Louis, Mo, US). Ethanol (EtOH), dimethylformamide (DMF), and acetone (ACE) of analytical grade were obtained from Merck KGaA (Darmstadt, Germany). Methanol (MeOH) and acetonitrile (ACN) of HPLC grade were purchased from Anaqua Global International Inc, Ltd (Cleveland, OH, US). Water was purified through a Direct-Q water purifier (Water Corp., Milford, MA, USA).

### 2.2. Preparation of Polymeric Drug Nanoparticles

**Figure 2:**
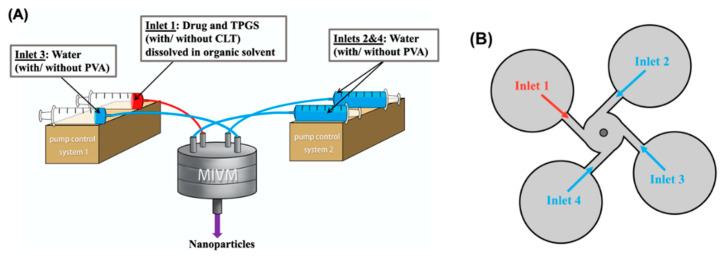
The (**A**) experimental set-up and (**B**) schematic of multi-inlet vortex mixer (MIVM).

All the polymeric drug nanoparticles were prepared by flash nanoprecipitation using a custom-made four-stream MIVM, the experimental set-up and geometry of which are shown in Figure 2. Briefly, the drug and TPGS with/without CLT were dissolved in an organic solvent for inlet 1, while the other streams consisting of water with/without 0.05% *w/v* PVA were served as a non-solvent(inlets 2–4). The detailed formulation of different nanoparticles is summarized in Table 2. The selection of the stabilization systems for the drug nanoparticles was based on the previous studies [27,28,29]. The flow rates of inlets 1 and 3 were controlled by syringe pump 1 and set at 10 mL/min, while those of inlets 2 and 4 were controlled by syringe pump 2 and set at 90 mL/min. The Reynold number (Re), which is used to characterize the flow pattern, of each formulation was calculated through the following equation:

(1) $$Re=\overset{4}{\underset{i=1}{\sum }}\frac{\rho_{i}v_{i}d}{\mu_{i}}=\frac{4}{\pi d}\overset{4}{\underset{i=1}{\sum }}\frac{\rho_{i}Q_{i}}{\mu_{i}}$$

where i is the stream number, d is the internal diameter of MIVM, ρ is the fluid density (kg/m^3^), μ is the fluid viscosity (kg/(m·s)), and Q is the stream flow rate (m^3^/s). Re of all formulations was >4000 to ensure an efficient mixing of organic and aqueous streams [19]. As a result, the drug nanoparticles with 5% *v/v* organic solvent were collected from the outlet of the MIVM.

### 2.3. Analysis of Particle Size and Particle Size Distribution

The z-average particle size and particle size distribution of nanoparticles were analyzed using a Delsa^TM^ Nano C analyzer (Beckman Coulter, Inc., Brea, CA, US) equipped with a dual 30 mW laser (with λ = 658 nm). Dynamic light scattering (DLS) was employed to measure the intensity of the light scattered by the nanoparticle in the suspension at a scattering angle of 165°. The polydispersity index (PDI) was obtained to represent the size distribution of the nanoparticle. The physical stability of the nanoparticle was monitored by the size change as a function of time. Nanoparticle formulations with an increase in z-average particle size by >20%, a PDI > 0.3, or the observation of a visible precipitant was considered as unstable.

### 2.4. Measurement of Zeta Potential

The zeta potential of the nanoparticle was also measured using the Delsa^TM^ Nano C analyzer by adding the sample into the high concentration cell equipped with a transparent electrode. The zeta potential reflected the electrokinetic potential at the slipping plane of the sample, which was derived from the electrophoretic mobility.

### 2.5. High Performance Liquid Chromatography (HPLC)

The concentrations of ITZ, VitD3, and FLU were determined using an HPLC equipped with a diode array detector (Agilent 1200 series, Agilent Technologies, Lexington, MA, US) and an Agilent Zorbax Eclipse Plus C18 column (5 µm, 250 mm × 4.6 mm). For ITZ, the mobile phase consisted of 60% *v/v* ACN and 40% *v/v* 10 mM ammonium acetate buffer (pH = 5.7) [30]. For VitD3, the mobile phase was 100% MeOH. For FLU, the mobile phase was a mixture of 70% *v/v* ACN and 30% *v/v*, 0.01% *v/v* trifluoroacetic acid (pH = 2.95). A 50-μL aliquot of each sample solution was injected and ran at a flow rate of 1 mL/min at room temperature. The detection wavelength was set as 261 nm, 265 nm, and 245 nm for ITZ, VitD3, and FLU, respectively. Calibration curves were constructed and the limit of detection (LOD) was determined as 0.09 μg/mL for ITZ (R^2^ = 0.999), 0.05 μg/mL for VitD3 (R^2^ = 1.000), and 0.02 μg/mL for FLU (R^2^ = 0.999).

### 2.6. Measurement of Drug Recovery During Filtration

Total of 0.1 mg/mL free drug solutions were filtered by 0.45 μm nylon syringe filters (Membrane-Solutions, Plano, TX, US), Acrodisc^®^ syringe filters with 0.2 μm Supor^®^ membrane (PALL, Port Washington NY, US), or ultrafiltered using Amicon® Ultra-15 centrifugal filters equipped with low-binding Ultracel® regenerated cellulose membrane with an molecular weight cut-off (MWCO) of 30 kDa (Merck KGaA, Darmstadt Germany). The solutions and the filtrates were collected for HPLC assay of drug concentration. The drug recovery (%) was calculated by the following equation:

(2) $$Drug\;recovery\left(\%\right)=\frac{conc.\;of\;filtrate}{conc.\;of\;solution}\times 100\%$$

### 2.7. Validation of Centrifugal Ultrafiltration

To validate the centrifugal speed and duration for centrifugal ultrafiltration using Amicon^®^ Ultra-15 centrifugal filters, the nanoparticles were centrifuged at designated speeds for different durations. The filtrate was collected for HPLC assay of free drug content and intensity check using DLS. The concentrate was resuspended in water for measurement of the particle size by DLS.

### 2.8. Determination of Drug Loading and Encapsulation Efficiency

The free drug and nanoparticles were separated through centrifugal ultrafiltration using Amicon^®^ Ultra-15 centrifugal filters. Briefly, 15 mL of fresh nanoparticles was loaded into the filter device and centrifuged at 4000× *g* for 20 min. The filtrate containing solely free drug was collected for HPLC assay, while the concentrate was freeze-dried. The freeze-dried powders were weighed and dissolved in an organic solvent for HPLC assay of the drug content in the nanoparticle. The drug loading (DL) and encapsulation efficiency (EE) were calculated using the following equations [20]:

(3) $$EE\;\left(\%\right)=\frac{total\;amount\;of\;drug-amount\;of\;free\;drug}{total\;amount\;of\;drug}\times 100\%$$

(4) $$DL\;\left(\%\right)=\frac{total\;amount\;of\;drug\;in\;nanoparticles}{total\;amount\;of\;nanoparticles}\times 100\%$$

### 2.9. Measurement of Drug Solubilities in Water and Release Medium

Excess amounts of ITZ, VitD3, and FLU were separately added in screw-capped test tubes with 3 mL of water and their respective release medium recommended by the US FDA [31]: 0.1 M HCl solution for ITZ; 0.1% SDS *w/v* solution for VitD3; phosphate buffered saline (PBS, pH = 7.4) for FLU. All the suspensions were shaken at room temperature for 72 h. Samples were withdrawn and filtered through 0.45-μm nylon syringe filters, followed by dilution to appropriate concentrations for HPLC assay.

### 2.10. Release Study under Sink Condition

The release studies of the nanoparticles were conducted in triplicates using both dialysis membrane (DM) and sample and separate (SS) methods. For DM method, 2.5 or 5 mL of nanoparticles with 1.25 mg drug was loaded into Spectra/Por^®^ 3 standard regenerated cellulose dialysis tubing (Repligen, Waltham, MA, US) with an MWCO of 3.5 kDa and clipped by standard closures (Repligen, Waltham, MA, US). The dialysis tube was immersed into 450 mL release medium at 37 °C with a magnetic stirrer stirring at 75 rpm. At 5, 15, 30, 45, 60, 75, 90, 120, 180, and 360 min, 3 mL of the dissolution medium was withdrawn and replaced with an equal volume of fresh medium. The sample solution was treated with EtOH to free the entrapped drug and assayed by HPLC for drug content. In addition, at 1 h and 3 h, the nanoparticles within the dialysis membrane (i.e., the donor compartment) were taken for measurements of intensity and particle size by DLS. Subsequently, the nanoparticles were centrifugally ultrafiltrated to obtain filtrate for HPLC assay of free drug content.

**Figure 3:**
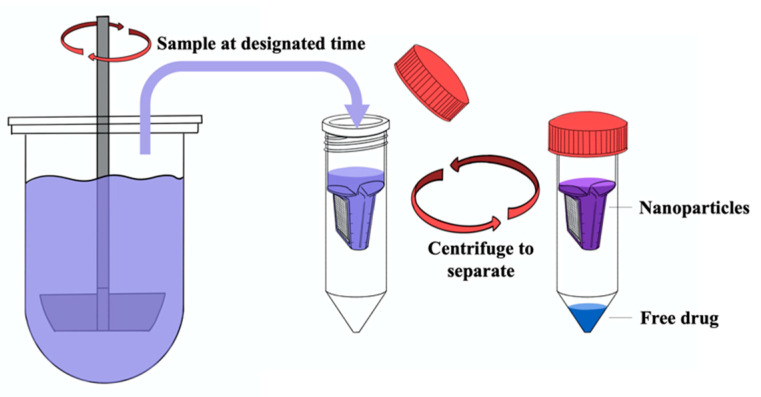
Procedures of SS + CU method.

For SS method, the Copley Dissolution Tester DIS8000 (Copley Scientific Limited, Nottingham, UK) was employed. Total of 5 or 10 mL of nanoparticles with 2.5 mg drug were added into the USP apparatus II (Paddle) with 900 mL release medium at 37 °C and a paddle rotation rate at 75 rpm. The nanoparticles and free drug were separated through 1) 0.45 μm nylon syringe filters (SS + 0.45 μm Filter), 2) Acrodisc^®^ syringe filters with 0.2 μm Supor^®^ membrane (SS + 0.2 μm Filter), or 3) centrifugal ultrafiltration using Amicon® Ultra-15 centrifugal filter equipped with low-binding Ultracel® regenerated cellulose membrane with an MWCO of 30 kDa (SS + CU). For the centrifugal ultrafiltration technique, 5 mL of the sample taken from the apparatus was loaded into the centrifugal unit and spun at 1000× *g* for 5 min (Figure 3). The filtrate was collected and further treated with EtOH to free the drug entrapped by the polymer. Subsequently, the drug content was assayed by the HPLC. The dissolution profiles of the raw drugs were also obtained using the same dissolution tester and testing conditions.

### 2.11. Mathematical Modeling of the Release Kinetics

The release data obtained by the DM and SS + CU methods were separately fitted to two mathematical models, i.e., Korsmeyer-Peppas model (Equation (5); [32]) and Baker-Lonsdale model (Equation (6); [33]). For Korsmeyer-Peppas model, only the data points with less than 60% release would be used for model fitting [32].

(5) $$\frac{M_{t}}{M_{\infty }}=k_{1}t^{n}$$

(6) $$f_{1}=\frac{3}{2}\left[1-{\left(1-\frac{M_{t}}{M_{\infty }}\right)}^{\frac{2}{3}}\right]-\frac{M_{t}}{M_{\infty }}=\frac{3D_{f}C_{fs}\varepsilon }{r_{0}^{2}C_{0}\tau }t=k_{2}t$$

where $M_{t}$ is the drug mass released at time t; $M_{\infty }$ is the total drug mass; k_1_ is a constant related to the structural and geometric characteristics of the dosage form; n is the release exponent indicating the release mechanism; $D_{f}$ is the diffusion coefficient; $C_{fs}$ is the drug solubility in the release medium; ε is the porosity of the dosage form; r_0_ is the radius of the dosage form; $C_{0}$ is the initial concentration of drug in the dosage form; τ is the tortuosity factor of the capillary system.

### 2.12. Statistical Analysis

All the experiments were conducted in triplicates. One-way ANOVA followed by Tukey’s test was employed for data analysis. A *p*-value less than 0.05 was considered statistically significant. Linear regression was used to determine the correlation between the physicochemical properties of the nanoparticles as well as the raw drug and their release kinetics.

## 3. Results and Discussion

### 3.1. Preparation and Characterization of Nanoparticles

Three different drug nanoparticle formulations with the z-average particle size less than 100 nm and PDI < 0.3 were prepared by using the MIVM. The PEGylated polymer, TPGS, was chosen as the primary stabilizer because of its amphiphilic and biodegradable natures. It is a GRAS-listed excipient for pharmaceutical formulations. ITZ, VitD3, and FLU were selected as the cargo because they possess various physicochemical properties (Table 1). Only FLU was slightly soluble in water (22.0 ± 5.7 μg/mL), while ITZ and VitD3 were practically water insoluble as their experimental solubilities were lower than the LOD of the HPLC (i.e., 0.09 μg/mL for ITZ and 0.05 μg/mL for VitD3). The stream flowrates remained the same for all sample preparation, while the formulation parameters such as organic solvent, initial drug concentration, drug-TPGS ratio, and co-stabilizer, varied in order to generate nanoparticles with adequate particle size (<100 nm) and stability for subsequent analysis. The preparation method of the ITZ and FLU nanoparticles were based on the previous research works [29,34].

Although the Z-average particle sizes of these nanoparticles were different, where the ITZ nanoparticle was the largest (93.75 ± 4.33 nm), the VitD3 nanoparticle was the second largest (41.62 ± 3.49 nm), and the FLU nanoparticle was the smallest (28.18 ± 4.22 nm), the PDIs of all the nanoparticles were below 0.3, indicating a narrow size distribution of each formulation (Figure S1). The ITZ nanoparticle exhibited a nearly null zeta potential, suggesting that most of the ITZ was encapsulated as the zeta potential would tend to be positive if ITZ was present on the surface of the nanoparticle. The zeta potential of the VitD3 nanoparticle was also neutral (Table 3). However, the FLU nanoparticle exhibited a negative zeta potential, which might be due to the presence of FLU on the surface. The FLU could be free FLU, since the EE of FLU nanoparticles was only 86.06 ± 1.22%, as well as the FLU presented on the surface of nanoparticles. The experimental DLs of all the nano-formulations were consistent to the theoretical values, whereas only ITZ and VitD3 were fully encapsulated by the stabilizers.

**Figure 4:**
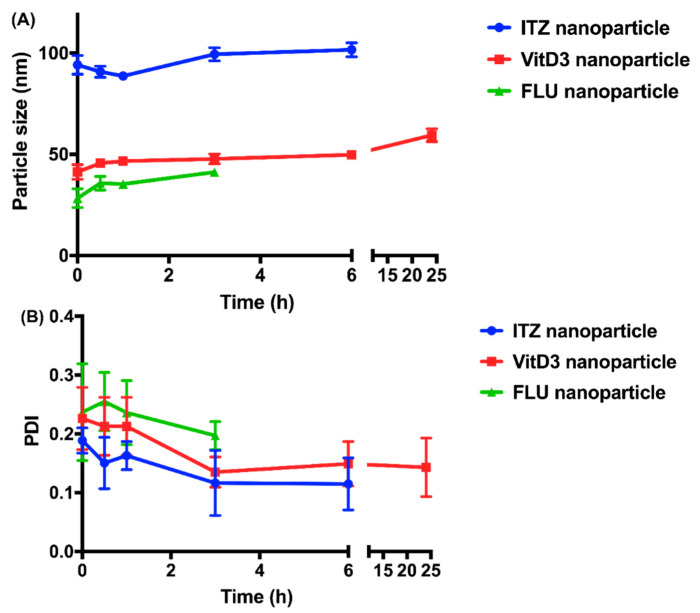
The time-course changes in (**A**) particle size (nm) and (**B**) polydispersity index (PDI) of nanoparticles stored at room temperature.

To examine the physical stability of all the nanoparticles, the particle size and PDI of the nanoparticles were regularly monitored. In order to ensure all the nanoparticles were stable prior to the release study, various co-stabilizers were employed. CLT was added in the organic stream during the preparation of ITZ nanoparticles and VitD3 nanoparticles because its highly hydrophobic nature could accelerate the molecular rearrangement process of amphiphilic polymer, leading to more hydrophilic block, Polyethylene glycol (PEG), present on the surface [27]. For the FLU nanoparticles, an aqueous solution containing 0.05% *w/v* PVA was employed as the antisolvent to increase the physical stability as the steric hindrance can be enhanced by the hydrophilic layer formed by PVA. In terms of the physical stability, VitD3 nanoparticles (7.00 ± 4.58 h) and FLU nanoparticles (0.67 ± 0.29 h) were limited by the increase of particle size, while that of ITZ nanoparticles (6.00 ± 0.00 h) were restricted by the observation of visible precipitants (Figure 4 and Table 3). Not surprisingly, the physical stability of nanoparticles was positively correlated with the lipophilicity of the entrapped drug (R^2^ = 0.910).

### 3.2. Validation of the Separation Methods

To minimize the analytical error, the drug loss during filtration and centrifugal ultrafiltration were determined before the experiments using these techniques. For all the drugs, there was no significant difference between the concentrations of the unprocessed solution and the filtered solutions (*p* > 0.05: 0.695 for ITZ, 0.752 for VitD3, 0.165 for FLU) and the absolute variations in assay were less than 0.5% (Table 4). Thus, the drug loss was considered negligible for subsequent data analysis. 

Although centrifugal ultrafiltration has been widely used to separate the free drug from nanoparticles, the impacts of centrifugal speed and duration on the stability of the nanoparticle remain unexplored and these could significantly affect the accuracy of the data obtained such as EE, DL, and even the release kinetics [20,35,36]. Since the FLU nanoparticle was the least stable among all the three formulations, it was selected as the model for the validation of the centrifugal ultrafiltration method. While the same volume of the nanoparticles was loaded into the filter device for all groups, they were centrifuged at different speeds and durations. The intensity of the light scattering in the filtrate was measured using DLS to access the efficiency of the centrifugal ultrafiltration under different processing conditions. The intensity of the light scattering in a solution or a suspension represented its light scattering strength and the light scattered by the ultra-purified water (UPW) was 62 ± 27 cps. Because the scattered light is closely related to the number and the size of the particles in sample, a higher intensity than that of the UPW could serve as an indicator of the presence of the nanoparticles in the filtrate [37]. In other words, if the intensity of the light scattering in the filtrate was significantly larger than UPW, it would imply an incomplete separation of the nanoparticles and the free drug.

**Figure 5:**
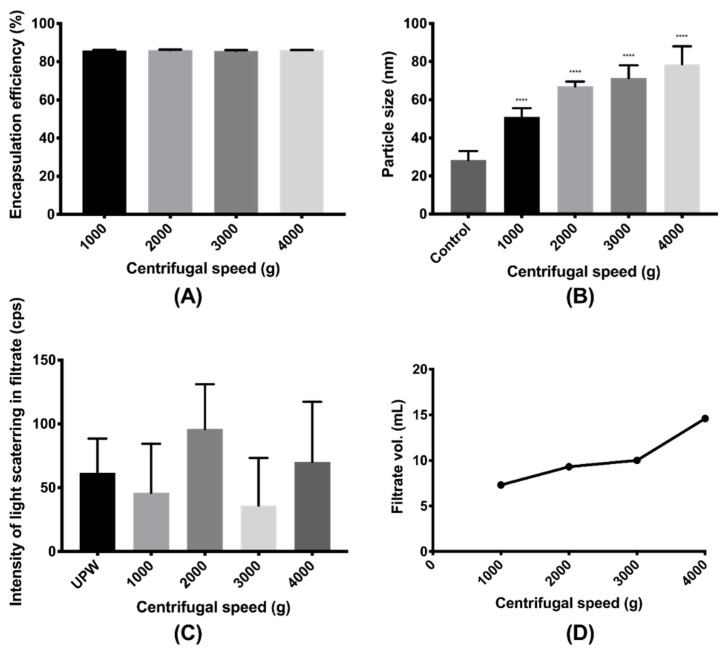
Effects of centrifugal speed on the (**A**) encapsulation efficiency and (**B**) particle size of the FLU nanoparticle as well as (**C**) intensity of light scattering in filtrate and (**D**) volume of the filtrate for duration of 20 min (**** denotes 0.0001 ≥ *p* comparing with control).

**Figure 6:**
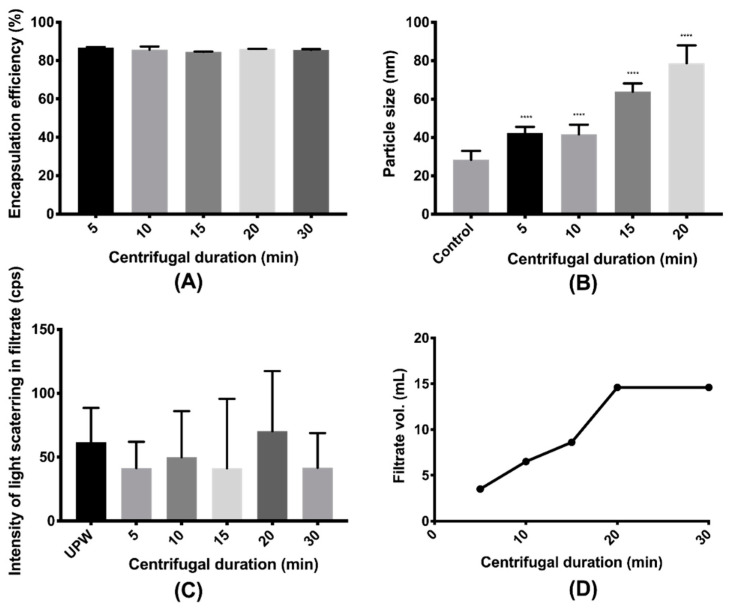
Effects of centrifugal duration on the (**A**) encapsulation efficiency and (**B**) particle size of the FLU nanoparticle as well as (**C**) intensity of light scattering in filtrate and (**D**) volume of the filtrate at speed of 4000× *g* (**** denotes 0.0001 ≥ *p* comparing with control).

When the centrifugal duration was fixed at 20 min, the centrifugal speed showed minimal impact on either the EE of the nanoparticle (*p* = 0.486 > 0.05, Figure 5A) or the intensity of the light scattering in the filtrate (*p* = 0.388 > 0.05, Figure 5C). On the other hand, when the centrifugal speed was fixed at 4000× *g*, neither the EE of the nanoparticle (*p* = 0.107 > 0.05, Figure 6A) nor the intensity of the light in the filtrate (*p* = 0.906 > 0.05, Figure 6C) was significantly altered with the rise of the centrifugal duration. This revealed no substantial damage to the nanoparticle caused by the centrifugal ultrafiltration when the speed and the duration increased. All the intensities of the filtrates obtained under varying speeds and durations were not significantly different from that of the UPW (*p* = 0.813 > 0.05), indicating an absence of nanoparticles in the filtrate. However, compared to the unprocessed nanoparticles, the particle sizes after centrifugal ultrafiltration were all larger (*p* < 0.0001). A trend was clearly observed that a higher centrifugal speed and a longer centrifugal duration could result in a larger particle size of the resuspended concentrate, which could be attributed to the aggregation of nanoparticles during centrifugation. When the nanoparticles were centrifuged at 4000× *g* for 30 min, precipitates were even observed. The volume of the filtrate was also increased with the increase of the centrifugal speed and duration, but the volume would remain the same if the sample was centrifuged at 4000× *g* for longer than 20 min. As the concentrate and the filtrate had the same concentration of the free drug, more volume of concentrate implied the presence of more amount of free drug with the nanoparticles. Thus, 15 mL of nanoparticles was centrifuged at 4000× *g* for 20 min for EE and DL determinations to ensure complete separation of the free drug and nanoparticles. Under such condition (Figures S2–S4), the EEs of both the ITZ nanoparticle and the VitD3 nanoparticle were determined as >99.96%. The intensities of the filtrates were not significantly different from that of the UPW (*p* = 0.789, 0.189 > 0.05), and the volumes of the filtrates collected from all the three nanoparticles were essentially the same as 14.6 mL. While the particle size of ITZ nanoparticles significantly increased to 136.4 ± 11.7 nm (*p* < 0.0001), the VitD3 nanoparticle only became a slightly larger, with a particle size of 45.7 ± 1.5 nm (*p* = 0.004 < 0.01).

For the release study, since only a limited volume of sample can be drawn from the release medium, 5 mL of sample will be taken for centrifugal ultrafiltration at 1000× *g* for 5 min. Under this condition (Figures S2–S4), 1.5 mL of the filtrate was collected for all the nanoparticles for HPLC assay of free drug content. The particle size of the FLU nanoparticle increased from 28.18 ± 4.22 nm to 66.0 ± 15.8 nm (*p* < 0.0001), and the particle size of the VitD3 nanoparticle also slightly increased from 41.62 ± 3.49 nm to 46.2 ± 2.7 nm (*p* = 0.002 < 0.01), while the particles size of the ITZ nanoparticle was not altered significantly (*p* = 0.568 > 0.05). However, the encapsulation efficiency of the nanoparticle and the intensity of the light in the filtrate still remained unchanged (*p* > 0.05), indicating no nanoparticles were damaged or penetrated through the filter membrane. Therefore, this condition was considered appropriate to separate the nanoparticles and the free drug during the in vitro release study.

### 3.3. Release Profiles of Nanoparticles

All the release studies were conducted under sink conditions because a non-sink condition could substantially underestimate the drug release from nanoparticles [38]. Hence, the solubilities of the drugs in their respective release medium were determined prior to the release study. The selection of release medium was based on the USP dissolution database, where 0.1 M HCl solution, 0.1% SDS *w/v* solution, and PBS (pH = 7.4) were employed as the release medium for ITZ, VitD3, and FLU, respectively [31]. ITZ was much less soluble than VitD3 and FLU in the release medium, while FLU was the most soluble (Table 1). According to their solubilities, different volumes of nanoparticles with equal amount of drug (i.e., 1.25 mg) were loaded into the dialysis tube with 450 mL release medium outside, and a double amount of nanoparticles was added in USP apparatus II (Paddle) with 900 mL release medium. The sink condition was maintained throughout the release studies. 

**Figure 7:**
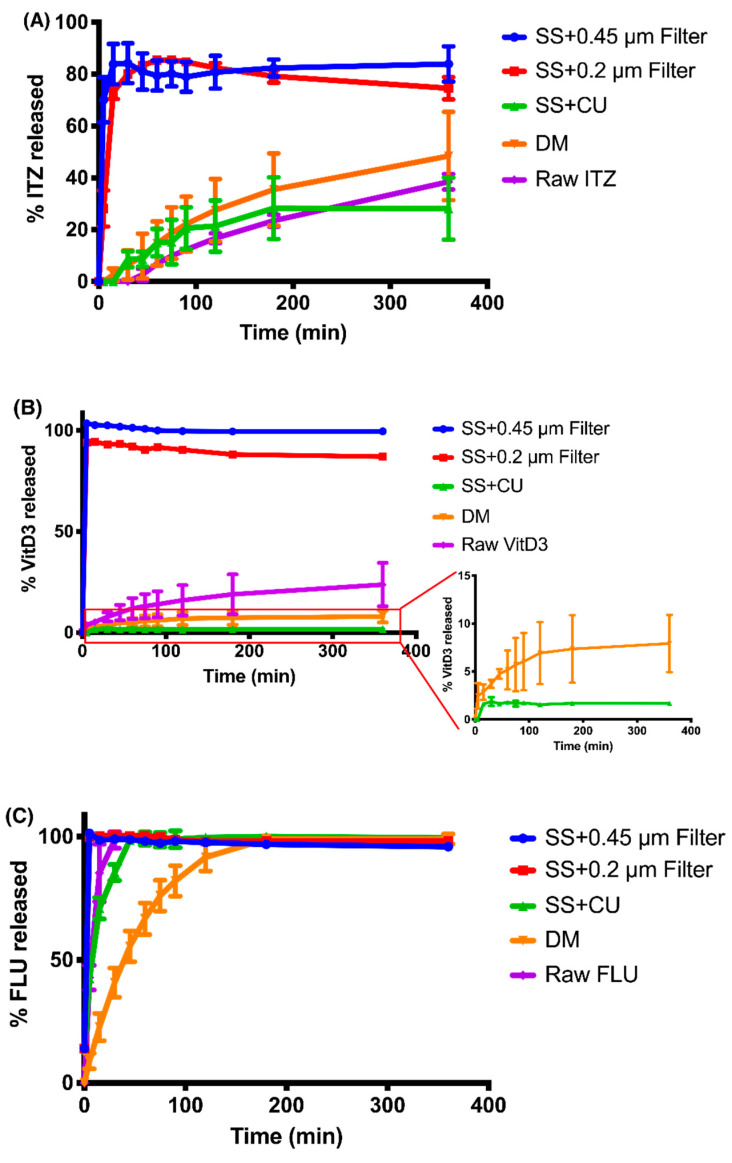
Release profiles of (**A**) ITZ nanoparticles, (**B**) VitD3 nanoparticles, and (**C**) FLU nanoparticles obtained by different techniques and the dissolution profile of raw (**A**) ITZ, (**B**) VitD3, and (**C**) FLU.

**Figure 8:**
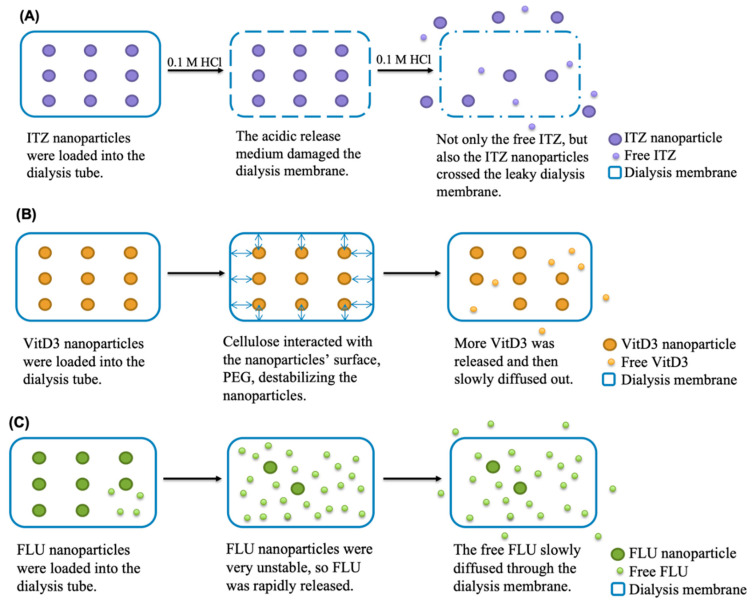
Dominant mechanisms of mis-estimated release kinetics of (**A**) ITZ nanoparticles, (**B**) VitD3 nanoparticles, and (**C**) FLU nanoparticles by the DM method.

The release profiles of ITZ nanoparticles and the dissolution profile of raw ITZ are plotted in Figure 7A. The SS methods using syringe filters (i.e., SS + 0.45 µm filter and SS + 0.2 µm filter methods) substantially overestimated the ITZ release owing to the incapability of separation of the free drug and the nanoparticles. As shown in Figure S1A, there was a small fraction of ITZ nanoparticles larger than 0.2 and 0.45 μm so that they could not pass through the filters, resulting in less than 100% ITZ release was observed in the release study. Although the fractions of ITZ released at 6 h were not significantly different between the SS method using centrifugal ultrafiltration (SS + CU) and DM method (*p* = 0.166 > 0.05), the time for commencement of ITZ release from nanoparticles varied. The release of ITZ started after 5 min when using the DM method but 15 min when using the SS + CU method. After 3 h, the release profile of ITZ nanoparticles determined by SS + CU plateaued at around 28%, while more ITZ was continuously released to the receiver compartment in the DM group. Therefore, compared to SS + CU, DM method showed a faster and larger ITZ release. Generally, DM method is considered to underestimate the drug release rate because the observed release rate of the drug in the acceptor compartment can be dictated by its diffusion rate across the dialysis membrane, which may mask the true release rate of the drug from the nanoparticles [13,14,37]. It could also be observed in our study that the concentrations of the free drug in the donor compartment were significantly higher than those in the receiver compartment at 1 h and 3 h (*p* < 0.0001) (Table S1). Even though, DM method appeared to overestimate the release kinetic of ITZ nanoparticles in the present study. This could be due to the leakage of nanoparticles induced by the low compatibility of the dialysis membrane against acidic conditions (Figure 8A). Hence, the intensity of the light scattering in the nanoparticles in the donor compartment had already been too low (1972 ± 195 cps) to detect the particle size after 1 h, implying only few nanoparticles left within the dialysis membrane (Table S1). While changing the release medium to PBS (pH = 7.4), the intensity of the light scattering in the nanoparticles in the dialysis tube remained as high as the original sample (12,752 ± 305 cps) and the particle size was increased to 104.8 ± 0.13 nm even after 6 h. In order to compare the repeatability of SS + CU and DM methods, the relative standard deviation (RSD) was calculated for each time point. The RSD of the release profile obtained by the SS + CU method was much lower than that determined by the DM method (*p* = 0.019 < 0.05), suggesting a higher repeatability of the SS + CU method.

As with the ITZ nanoparticle, the syringe filters cannot effectively separate the VitD3 nanoparticles, causing a pseudo burst release of VitD3 (Figure 7B). As no VitD3 nanoparticles were beyond 0.45 μm, but a few of the nanoparticles were larger than 0.2 μm, VitD3 displayed a complete release for the case of using 0.45 μm filters but only around 90% using 0.2 μm filters (Figure S1B). For the DM method, the initial VitD3 release from the nanoparticles was observed at 5 min. Subsequently, VitD3 was released slowly, attaining around 7.9% of release after 6 h. For the SS + CU method, the nanoparticle started drug release at 15 min. Meanwhile, the release kinetic of VitD3 nanoparticles also plateaued at approximately 1.7%, which was significantly lower than that of the DM group (*p* < 0.0001). Hence, both the release rate and the fraction of release determined by the DM method were significantly higher than that of the SS + CU group. Since the centrifugal ultrafiltration technique has been validated to ensure no leakage of drug from nanoparticles and no drug absorbed into the filter membrane, the SS + CU should not misestimate the release kinetic of VitD3 nanoparticles. In other word, the DM method overestimated the release kinetic of the nanoparticles once again, probably because of the extra damage to the nanoparticles. Previous studies found that cellulose could interact with PEG so that the nanoparticles within the dialysis membrane tended to accumulate to the surface of the dialysis membrane gradually during the release study, causing particle aggregation and subsequent destabilization (Figure 8B) [39,40]. As a result, the VitD3 nanoparticles in the donor compartment became unstable after 3 h with a PDI > 0.3, and more VitD3 was released to the receiver compartment (Table S1). Regarding the RSD, the SS + CU group were again much lower than those of the DM group (*p* < 0.0001).

As shown in Figure 7C, both 0.45 and 0.2 μm filters could not separate free FLU and FLU nanoparticles because of the relatively smaller particle size of FLU nanoparticles (Figure S3C). A complete release of FLU nanoparticles thus was seen at 5 min. Unlike the ITZ nanoparticle and the VitD3 nanoparticle, FLU nanoparticles released rapidly at the very initial stage regardless of the method used. To compare the release profiles determined by DM and SS + CU methods, the initial release rate (IRR) was calculated by the following equation:

(7) $$IRR=\frac{{\left(d_{m}/d_{t}\right)}_{initial}}{A}$$

where ${\left(d_{m}/d_{t}\right)}_{initial}$ is the slope of the initial linear region of the cumulative release curve and *A* is the specific surface area of the nanoparticles. As the same FLU nanoparticle formulation was used, the IRR ratio of the SS + CU group to the DM group could be simply obtained by the ratio of their slope, which was 3.3, implying the initial release rate of the SS + CU group was 3.3 times faster than the DM group. Since the FLU nanoparticle became unstable at room temperature within 1 h, it was questionable that the free FLU was gradually released from the nanoparticle over 3 h under a sink condition. In addition, when using the DM method, the average fraction of FLU release observed at 5 min was below 14%, which was the amount of free FLU already existing in the nanoparticles. According to the Table S1, after 1 h of the release study, all the FLU has already been released out, but the majority of them still stayed within the donor compartment. All of these suggested that the dialysis membrane substantially delayed the translocation of the released FLU to the sampling compartment, leading to an underestimated release kinetic obtained by the DM method (Figure 8C). On the contrary, the SS + CU group showed a rapid initial release followed by a plateau of 100% after 45 min, which correlated well with the physical stability of the FLU nanoparticle.

In addition to the release profiles, mathematical modeling was applied to study the release kinetics obtained by the DM and SS + CU methods. In this study, two mathematical models including Korsmeyer-Peppas model and Baker-Lonsdale model were selected for comparative analysis, which were commonly used for mathematical modeling of drug release from polymeric nanoparticles [41,42]. The former is commonly employed to study the release kinetics of polymeric dosage forms without known mechanisms. The release exponent (n) is used to characterize the release mechanism, where n = 0.43 represents a Fickian diffusion and 0.43 < n < 0.85 indicates a non-Fickian transport for spherical systems [43]. It should be noted that only data points with less than 60% release can be used for model fitting. The latter model is derived from the Higuchi model for spherical polymeric systems [44]. As shown in the Table 5, both models fitted all the data well with high coefficients of determination (R^2^ > 0.9). Interestingly, the release exponents of the DM groups were all larger than those of the SS + CU groups, indicating the release kinetics determined by the SS + CU method were more prone to follow the quasi Fickian or Fickian diffusion. This was because the diffusion of drug from the core to the particle surface required penetration through the stabilizers. However, it is worth mentioning that both Korsmeyer-Peppas model and Baker-Lonsdale model were empirical models primarily developed to explain the drug release from conventional controlled release dosage forms [42]. A more suitable empirical model for release kinetics of nanoparticles should be developed based on the release data collected by SS + CU method accordingly.

It should be noted that the release rates of drug-loaded polymeric nanoparticles were not always faster than the dissolution rates of raw drugs (Figure 7). Among the three drugs, FLU had the highest dissolution rate and fraction dissolved, while VitD3 possessed faster dissolution rate than ITZ. Such that, the dissolution rate was positively associated with the solubility of the raw drug in the release medium (R^2^ = 0.999). On the other hand, the release rates and fractions of drug released followed the descending order as FLU > ITZ > VitD3, which were reversely related to their physical stability. The nanoparticles possessing higher physical stability exhibited a lower release rate (R^2^ = 0.995) and fraction of release (R^2^ = 0.993). In addition, the fractions of drug released were also negatively correlated to the lipophilicity of the model drugs (R^2^ = 0.951). Therefore, rather than improving the solubility of the hydrophobic drugs, stable nanoparticles were more prone to protect the loaded cargos to achieve a sustained release.

### 3.4. Significance of the Study

Based on the above findings, the newly developed SS + CU method appeared to be a more accurate and reproducible approach to study the in vitro release kinetics of polymeric nanoparticles with different particle sizes and encapsulated drugs. USP apparatus II is a standardized equipment with controlled temperature, rotary speed, etc., while conditions under the DM method usually differ among the research teams regarding the type of the dialysis membrane and closures, volume of medium, etc., [12,13,14]. Even if the stirring speed is the same, the size of the stirrer might cause significant variation in stirring strength. In addition, the shape of the dialysis tube as well as the distance between the dialysis tube and the stirrer were hard to be controlled for different samples. This was observed in our study as the RSDs of all the DM groups were much higher than other groups. In the real situation when the nanoparticles are administered into the body, they are distributed in the body fluid but not stay together within a thin film like dialysis membrane so the USP apparatus II is apparently more suitable to mimic the in vivo condition. In regard to the pitfalls of DM method, diffusion of free drug across the dialysis membrane can mask the actual release rate (case of the FLU nanoparticle); the dialysis membrane can be vulnerable in some release media like strong acids and organic solvents (case of the ITZ nanoparticle); dialysis membrane can interact with polymers like PEG to destabilize the nanoparticles (case of the VitD3 nanoparticle); the closure may not be completely tight to ensure null leakage of nanoparticles; some highly lipophilic drugs may partition into the dialysis membrane; and the high local concentration within the dialysis tube may hamper the stability of the nanoparticles. Although the SS + CU method showed its merits in the in vitro release study of nanoparticles, the filter membrane of the centrifugal unit is not compatible with high concentrations of surfactants and organic solvents [45], thus the release medium should be carefully chosen.

Nowadays, IVIVC has been an active area for pharmaceutical development as it can facilitate the optimization of the formulations with the fewest possible trials in animals and humans. The critical step of establishing an IVIVC is to obtain a proper in vitro release profiles. Therefore, with the assistance of this novel SS + CU method, a more accurate and precise in vitro release kinetics of the polymeric nanoparticle can be easily obtained, achieving better correlation between the in vitro data and the in vivo response.

## 4. Conclusions

Through the comparative study of the release kinetics of three different drug nanoparticles in the sink condition by using four different methods, the SS + CU with optimized centrifugal speed and duration was demonstrated to be the most appropriate to reflect the actual in vitro release performance of nanoparticles. It was found that the SS method using syringe filters could not separate the free drug from the nanoparticles and resulted in a pseudo burst release of drugs. On the other hand, the DM method appeared to misestimate the release kinetics of nanoparticles through separate mechanisms that depend on the stability and the surface properties of the nanoparticles as well as the release medium. With this novel method of in vitro release study, we expect it will facilitate the development of in vitro-in vivo correlation (IVIVC) for nanoparticle-based drug delivery systems, thus reducing the regulatory burden and accelerating their clinical translation in near future.

## Figures and Tables

**Table 1:** The physicochemical properties of model drugs.

Drug	pK_a_	Log *P*	Aqueous Solubility (µg/mL)	Solubility in Release Medium (µg/mL)
ITZ	3.7 [21]	5.7 [22]	<0.09 *	8.6 ± 0.3
VitD3	-	7.5 [23]	<0.05 *	90.3 ± 6.7
FLU	4.5 [24]	4.2 [25]	22.0 ± 5.7	674.5 ± 4.9

* The limit of detection (LOD) of HPLC.

**Table 2:** The formulation parameters of nanoparticles.

Formulation	Drug	Initial Drug Conc. * (mg/mL)	Initial TPGS Conc. * (mg/mL)	Co-Stabilizer	Initial Co-Stabilizer Conc. (mg/mL)	Organic Solvent
ITZ nanoparticle	ITZ	5	5	CLT	1 *	DMF
VitD3 nanoparticle	VitD3	10	5	CLT	1 *	EtOH
FLU nanoparticle	FLU	10	20	PVA	0.5	ACE

* The concentration in the organic stream.

**Table 3:** The physicochemical properties of nanoparticles.

Formulation	Size (nm)	PDI (Polydispersity Index)	Zeta Potential (mV)	Drug Loading (%)	Encapsulation Efficiency (%)	Physical Stability (h)
ITZ nanoparticle	93.75 ± 4.33	0.19 ± 0.02	−0.87 ± 0.1	42.88 ± 0.78	>99.96 *	6.00 ± 0.00
VitD3 nanoparticle	41.62 ± 3.49	0.23 ± 0.05	−0.31 ± 0.22	65.72 ± 1.23	>99.98 *	7.00 ± 4.58
FLU nanoparticle	28.18 ± 4.22	0.26 ± 0.01	−61.23 ± 3.09	23.45 ± 0.99	86.06 ± 1.22	0.67 ± 0.29

* Calculated based on the LOD of HPLC.

**Table 4:** Drug recovery (%) during filtration and centrifugal ultrafiltration.

Drug	0.45 μm Filter	0.2 μm Filter	Amicon^®^ Ultra-15
ITZ	99.79 ± 0.14	99.86 ± 0.07	100.25 ± 1.01
VitD3	99.96 ± 0.04	99.90 ± 0.02	99.82 ± 0.37
FLU	100.44 ± 0.56	99.9 ± 30.47	99.78 ± 0.23

**Table 5:** The value of R^2^ and exponent (n) for modeling of release kinetics.

Formulation	Korsmeyer-Peppas Model		Baker-Lonsdale Model
	R^2^	n	R^2^
**ITZ nanoparticles**			
DM	0.987	0.680	0.994
SS + CU	0.939	0.510	0.902
**VitD3 nanoparticles**			
DM	0.985	0.290	0.995
SS + CU	0.966	0	0.989
**FLU nanoparticles**			
DM	0.999	0.816	0.995
SS + CU	0.994	0.465	0.991

## Supplementary Materials

The following are available online at https://www.mdpi.com/1999-4923/12/8/732/s1, Figure S1: Particle size distribution of the three nanoparticles; Figure S2: the change of encapsulation efficiency and particle size of the ITZ nanoparticles as well as intensity of light scattering in filtrate and volume of the filtrate under the centrifugal condition 1 (15 mL of nanoparticles at 4000*g* for 20 min) and the centrifugal condition 2 (5 mL of nanoparticles at 1000*g* for 5 min); Figure S3: the change of encapsulation efficiency and particle size of the VitD3 nanoparticles as well as intensity of light scattering in filtrate and volume of the filtrate under the centrifugal condition 1 (15 mL of nanoparticles at 4000*g* for 20 min) and the centrifugal condition 2 (5 mL of nanoparticles at 1000*g* for 5 min); Figure S4: the change of encapsulation efficiency and particle size of the FLU nanoparticles as well as intensity of light scattering in filtrate and volume of the filtrate under the centrifugal condition 1 (15 mL of nanoparticles at 4000*g* for 20 min) and the centrifugal condition 2 (5 mL of nanoparticles at 1000*g* for 5 min);Table S1: detailed information during the release studies using DM method.

## List of Abbreviations

**Abbreviation**	**Meaning**
**ACE**	Acetone
**ACN**	Acetonitrile
**CLT**	Cholesterol
**CF**	Continuous flow
**CU**	Centrifugal ultrafiltration
**DL**	Drug loading
**DLS**	Dynamic light scattering
**DM**	Dialysis membrane
**DMF**	Dimethylformamide
**EE**	Encapsulation efficiency
**EtOH**	Ethanol
**FLU**	Flurbiprofen
**HPLC**	High performance liquid chromatography
**ITZ**	Itraconazole
**IVIVC**	In vitro-in vivo correlation
**LOD**	Limit of detection
**MeOH**	Methanol
**MIMV**	Multi-inlet vortex mixer
**PDI**	Polydispersity index
**RSD**	Relative standard deviation
**SDS**	Sodium dodecyl sulfate
**SS**	Sample and separate
**TPGS**	D-α-tocopheryl polyethylene glycol 1000 succinate
**UPW**	Ultra-purified water
**VitD3**	Cholecalciferol
