# Supplementary material for: In Vitro Release Study of the Polymeric Drug Nanoparticles: Development and Validation of a Novel Method
Source: Pharmaceutics. 2020 Aug 4;12(8):732. doi: 10.3390/pharmaceutics12080732 (PMC7465254; doi:10.3390/pharmaceutics12080732)
Supplement: Supplementary file 1 [file pharmaceutics-12-00732-s001.pdf]

pharmaceutics

MDPI

Supplementary Materials: *In Vitro* Release Study of The Polymeric Drug Nanoparticles: Development and Validation of a Novel Method

Jingwen Weng, Henry H.Y. Tong and Shing Fung Chow

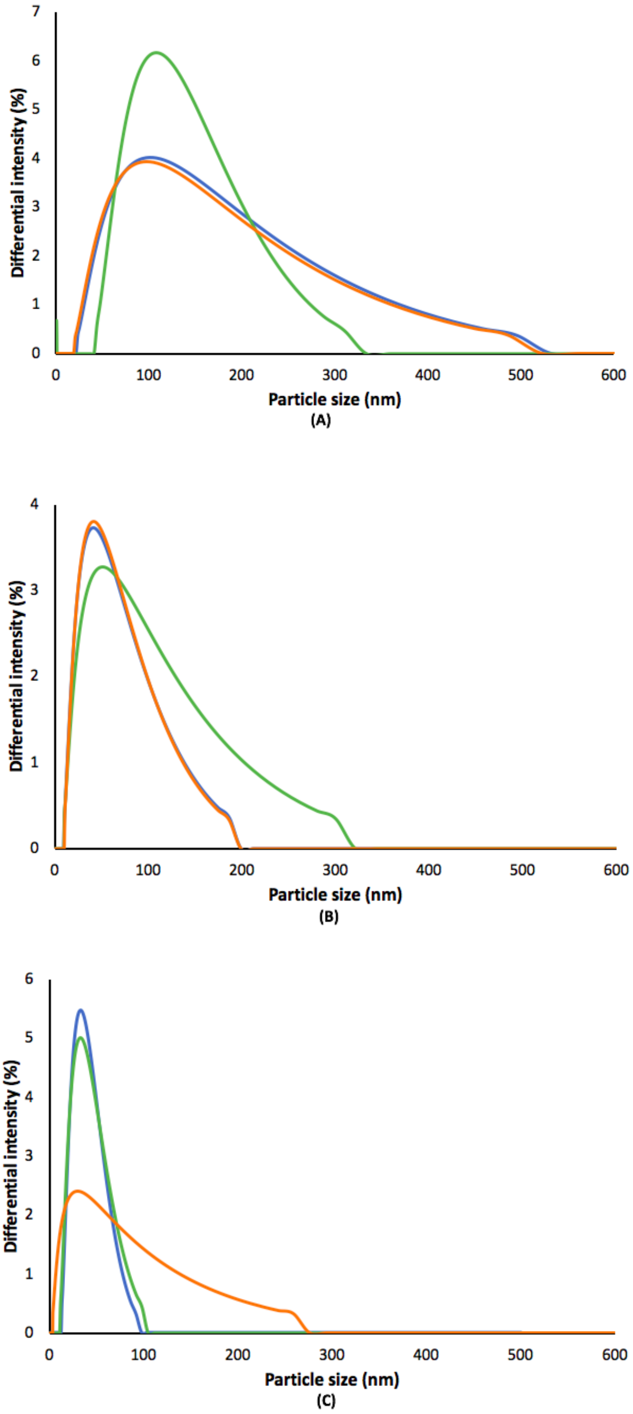

Figure S1. Particle size distributions of (**A**) ITZ (itraconazole) nanoparticles, (**B**) VitD3 (cholecalciferol) nanoparticles and (**C**) FLU (flurbiprofen) nanoparticles (n = 3).

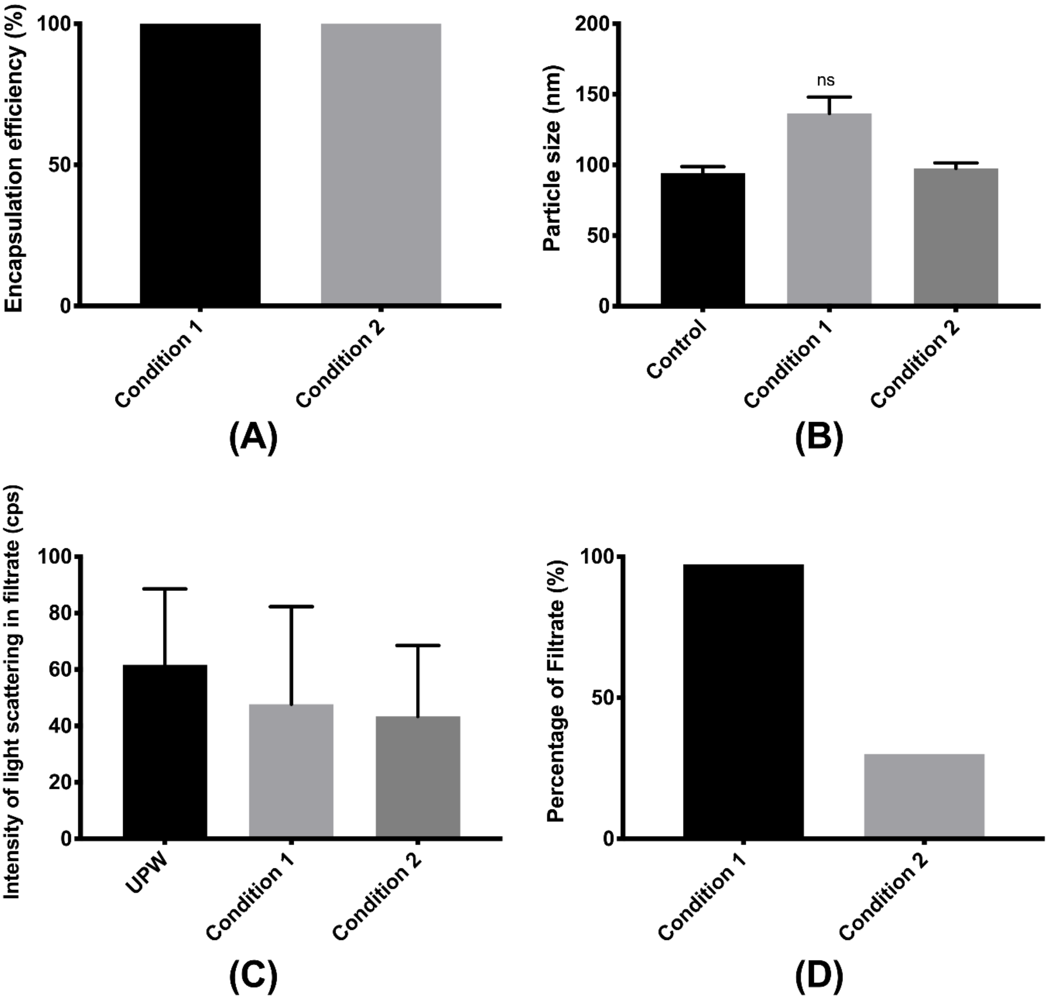

Figure S2. The change of (**A**) encapsulation efficiency and (**B**) particle size of the ITZ nanoparticle as well as (**C**) intensity of light scattering in filtrate and (**D**) percentage of the filtrate under the centrifugal condition 1 (15 mL of ITZ nanoparticles at 4,000g for 20 min) and the centrifugal condition 2 (5 mL of ITZ nanoparticles at 1,000g for 5 min) (ns—not significant).

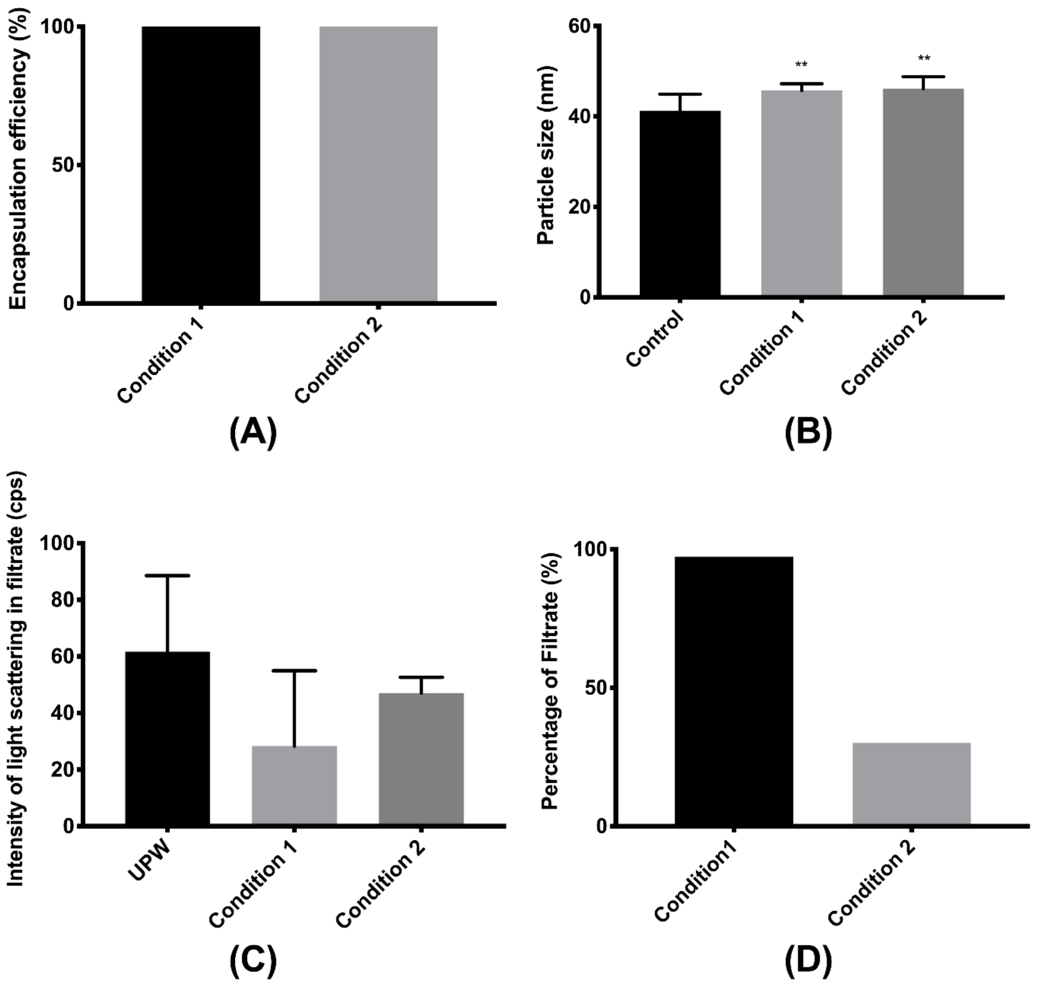

Figure S3. The change of (**A**) encapsulation efficiency and (**B**) particle size of the VitD3 nanoparticle as well as (**C**) intensity of light scattering in filtrate and (**D**) percentage of the filtrate under the centrifugal condition 1 (15 mL of VitD3 nanoparticles at 4,000g for 20 min) and the centrifugal condition 2 (5 mL of VitD3 nanoparticles at 1,000g for 5 min) (** denotes $0.01 \geq p > 0.001$).

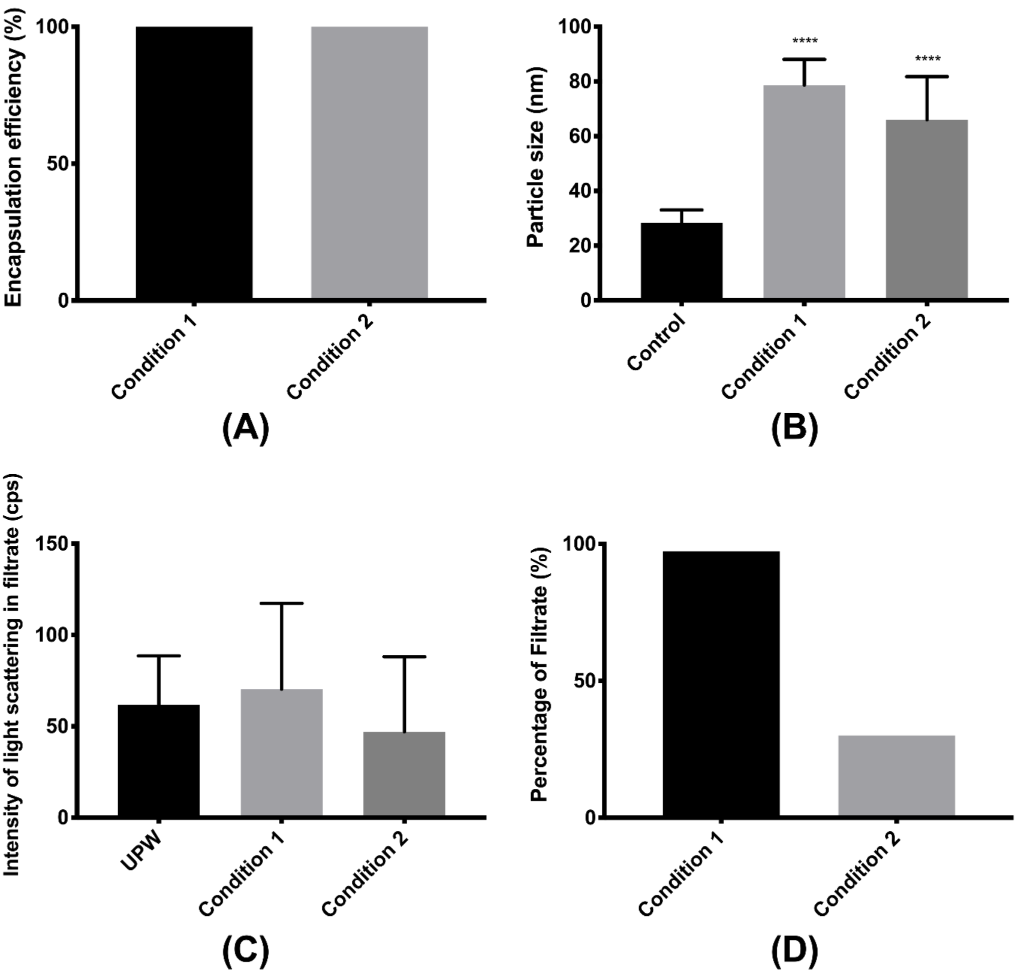

Figure S4. The change of (**A**) encapsulation efficiency and (**B**) particle size of the FLU nanoparticle as well as (**C**) intensity of light scattering in filtrate and (**D**) percentage of the filtrate under the centrifugal condition 1 (15 mL of FLU nanoparticles at 4,000g for 20 min) and the centrifugal condition 2 (5 mL of FLU nanoparticles at 1,000g for 5 min) (**** denotes $0.0001 \geq p$ comparing with control).

Table S1. Detailed information during the release studies using DM (dialysis membrane) method.

Formulation	Conc. of free drug in donor compartment (μg/mL)	Conc. of free drug in receiver compartment (μg/mL)	Intensity of light scattering in nanoparticles (cps)	Particle size (nm) & PDI (polydispersity index) in donor compartment
ITZ nanoparticle	At 1h<br>4.26 ± 0.49	0.41 ± 0.19	1,972 ± 195	undetectable
	At 3h<br>5.84 ± 0.13	0.99 ± 0.32	1,243 ± 361	-
VitD3 nanoparticle	At 1h<br>1.43 ± 0.32	0.14 ± 0.06	9,168 ± 288	39.95 ± 2.69<br>(0.23 ± 0.03)
	At 3h<br>1.20 ± 0.17	0.20 ± 0.09	10,226 ± 686	46.00 ± 2.37<br>(0.31 ± 0.03)
FLU nanoparticle	At 1h<br>172.80 ± 20.47	1.83 ± 0.18	860 ± 110	undetectable
	At 3h<br>17.62 ± 0.66	2.72 ± 0.5	736 ± 326	undetectable

© 2020 by the authors. Submitted for possible open access publication under the terms and conditions of the Creative Commons Attribution (CC BY) license (http://creativecommons.org/licenses/by/4.0/).
